# Key Factors in Decision Making for ECLS: A Binational Factorial Survey

**DOI:** 10.1177/0272989X211040815

**Published:** 2021-10-23

**Authors:** Daniel Drewniak, Giovanna Brandi, Philipp Karl Buehler, Peter Steiger, Niels Hagenbuch, Sabine Stamm-Balderjahn, Liane Schenk, Ana Rosca, Tanja Krones

**Affiliations:** Institute of Biomedical Ethics and History of Medicine, University of Zurich, Zurich, Switzerland; IInstitute of Intensive Medicine, University Hospital of Zurich, Zurich, Switzerland; IInstitute of Intensive Medicine, University Hospital of Zurich, Zurich, Switzerland; IInstitute of Intensive Medicine, University Hospital of Zurich, Zurich, Switzerland; Institute of Biomedical Ethics and History of Medicine, University of Zurich, Zurich, Switzerland; IInstitute of Medical Sociology and Rehabilitation Science, Charité–Universitätsmedizin Berlin, Berlin, Germany; IInstitute of Medical Sociology and Rehabilitation Science, Charité–Universitätsmedizin Berlin, Berlin, Germany; Institute of Biomedical Ethics and History of Medicine, University of Zurich, Zurich, Switzerland; IInstitute of Biomedical Ethics and History of Medicine, Clinical Ethics Unit, University Hospital Zürich, University of Zurich, Zurich, Switzerland

**Keywords:** extracorporeal life support, decision making, factorial survey, age, neurological status

## Abstract

**Background:**

Extracorporeal life support (ECLS) provides support to patients with cardiopulmonary failure refractory to conventional therapy. While ECLS is potentially life-saving, it is associated with severe complications; decision making to initiate ECLS must, therefore, carefully consider which patients ECLS potentially benefits despite its consequences.

**Objective:**

To answer 2 questions: First, which medically relevant patient factors influence decisions to initiate ECLS? Second, what are factors relevant to decisions to withdraw a running ECLS treatment?

**Methods:**

We conducted a factorial survey among 420 physicians from 111 hospitals in Switzerland and Germany. The study included 2 scenarios: 1 explored willingness to initiate ECLS, and 1 explored willingness to withdraw a running ECLS treatment. Each participant responded to 5 different vignettes for each scenario. Vignettes were analyzed using mixed-effects regression models with random intercepts.

**Results:**

Factors in the vignettes such as patients’ age, treatment costs, therapeutic goal, comorbidities, and neurological outcome significantly influenced the decision to initiate ECLS. When it came to the decision to withdraw ECLS, patients’ age, days on ECLS, criteria for discontinuation, condition of the patient, comorbidities, and neurological outcome were significant factors. In both scenarios, patients’ age and neurological outcome were the most influential factors.

**Conclusions:**

This study provided insights into physicians’ decision making processes about ECLS initiation and withdrawal. Patients’ age and neurological status were the strongest factors influencing decisions regarding initiation of ECLS as well as for ECLS withdrawal. The findings may contribute to a more refined understanding of complex decision making for ECLS.

## Introduction

Extracorporeal life support (ECLS) and extracorporeal membrane oxygenation (ECMO) are methods to provide mechanical support to patients with cardiopulmonary failure by a modified heart–lung machine. In the following, the comprehensive term *ECLS*, which includes all forms of heart and lung support, is used. There are 2 variations of ECLS: venovenous (VV) ECLS, which provides lung support only, and venoarterial (VA) ECLS, which supports both the heart and lungs.^1^ ECLS is not a cure but can sustain someone in emergency situations,^2^ specifically in cardiac^3^ and respiratory settings.^4–6^

ECLS is potentially lifesaving, but it is highly invasive and associated with severe complications, including hemorrhage, thromboembolism, and neurological complications.^7^ The increasing availability^8^ and belief that ECLS has become safer^9^ have generated several ethical questions,^10–12^ such as which patients should receive ECLS treatment, what the duration of ECLS support should be, and in what cases ECLS support should be discontinued.

Decision making to initiate ECLS should aim to select patients for whom ECLS is potentially beneficial. However, ECLS is a complex intervention, time sensitive, and dependent on the capacity of the medical centers in terms of both expertise and resources. Moreover, ECLS is an emerging technology, and current, evidence-based guidelines do not always apply to the specific, complex, clinical reality of individual situations.^13^ Timely, demanding decisions in clinically uncertain circumstances require heuristic short-cuts. While such “rules of thumb” allow clinicians to make quick decisions, they lack rigor; precision and accuracy must be weighed against speed and ease.^14^

Given these considerations, there is a growing need to better understand the decision making processes to initiate and to withdraw ECLS in adult patients. The goal of the present study was to answer 2 questions: First, which medically relevant patient factors influence decisions to initiate ECLS? Second, what are factors relevant to decisions to withdraw a running ECLS treatment?

## Methods

### Study Design

**Figure 1:**
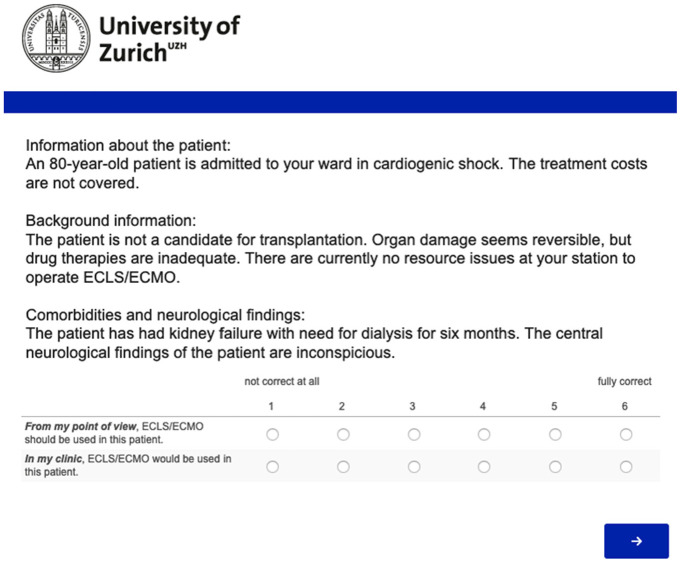
Example of a vignette presented to study participants (translated to English). The survey was conducted in German, French, and Italian. ECLS, extracorporeal life support.

A factorial survey was conducted that presented vignettes designed to explore physicians’ ELCS initiation and withdrawal decision making.^15,16^ Each vignette consisted of factors that were varied along a spectrum; for age, for example, there were 7 variations ranging from 35 to 90, or, for costs, for example, there were 2 variations: covered or not covered. In vignettes addressing initiation, there were 7 varying factors, and in the vignettes addressing withdrawal, there were 8 varying factors (Table 1 and Table 2). All variations of all the factors could be combined with each other. This resulted in 5 * 2 * 2 * 3 * 2 * 4 * 3 = 1,440 vignettes for the initiation scenario and 5 * 2 * 3 * 4 * 3 * 5 * 3 * 3 = 16,200 for the withdrawal scenario. Figure 1 shows an example of a vignette presented to the respondents.

**Table 1:** Factors Included in the Initiation Vignettes

Factor	Level	Vignette Wording
Information regarding the patient		
		A . . .
Age	35	. . . 35-year-old
	60	. . . 60-year-old
	70	. . . 70-year-old
	80	. . . 80-year-old
	90	. . . 90-year-old
		patient is admitted to your ward . . .
ECLS circuit	VV-ECLS/ECMO	. . . with severe ARDS.
	VA-ECLS/ECMO	. . . in cardiogenic shock.
Treatment costs	Covered	The treatment costs are covered.
	Not covered	The treatment costs are not covered.
Background information		
Therapeutic goal (bridge to)	Decision	The cause of the lung/heart failure is unknown and a final treatment plan has not yet been established.
	Recovery	The patient is not a transplant candidate. The organ damage appears to be reversible, but drug therapies are inadequate.
	Transplant	The patient is urgently listed for a lung/heart transplant (U).
Resources	No resource problem	There are currently no problems with the resources to operate ECLS/ECMO on your ward.
	Resource scarcity	There is currently a shortage of beds on your ward.
Comorbidities and neurological findings		
Comorbidities	No comorbidities	There are no other comorbidities.
	Kausch-Whipple operation with curative intent due to a pancreas CA 6 months ago.	Six months ago, a Kausch-Whipple operation with curative intent was performed on the patient due to a pancreas CA.
	Solitary metastatic colon carcinoma	The patient has a solitary metastatic colon carcinoma.
	Kidney failure with need for dialysis	The patient has had kidney failure requiring dialysis for 6 months.
Neurological outcome	Inconspicuous neurological findings	The patient’s central neurological findings are normal.
	Neurological damage possible	Severe central neurological damage cannot be ruled out.
	Neurological damage certain	Severe central neurological damage is assumed to be certain.

ARDS, acute respiratory distress syndrome; CA, carcinoma; ECLS, extracorporeal life support; ECMO, extracorporeal membrane oxygenation; VA, venoarterial; VV, venovenous.

**Table 2:** Factors Included in the Withdrawal Vignettes.

Factor	Level	Vignette Wording
Information regarding the patient		
		This is a . . .
Age in years	35	. . . 35-year-old
	60	. . . 60-year-old
	70	. . . 70-year-old
	80	. . . 80-year-old
	90	. . . 90-year-old
		patient who was admitted to a . . .
ECLS circuit	VV-ECLS/ECMO	. . . VV-ECLS/ECMO due to severe ARDS . . .
	VA-ECLS/ECMO	. . . VA-ECLS/ECMO due to cardiogenic shock . . .
ECLS duration in days	2	. . . 2 days ago.
	7	. . . 7 days ago.
	14	. . . 14 days ago.
	21	. . . 21 days ago.
	40	. . . 40 days ago.
Criteria for withdrawal	Not defined	No criteria for ECLS/ECMO withdrawal have been defined.
	Defined but not fulfilled	Withdrawal criteria were defined before ECLS/ECMO was started. However, these have not yet been fulfilled.
	Defined and fulfilled	Withdrawal criteria were defined before ECLS/ECMO was started. The criteria are fulfilled.
Condition of the patient	Improved	The patient’s condition has improved since ECLS/ECMO was started.
	Worsened	The patient’s condition has worsened since ECLS/ECMO was started.
	Unchanged	The patient’s condition has not changed since ECLS/ECMO was started.
Background information		
Therapeutic goal (bridge to)	Decision	The initial indication for ECLS/ECMO therapy was to determine the cause of the lung/heart failure and to define a final therapy concept for the patient.
	Recovery	The patient is not a transplant candidate. The organ damage appeared to be reversible, but drug therapies were inadequate.
	Transplant	The patient is urgently listed for a lung/heart transplant (U).
Comorbidities and neurological findings		
Comorbidities	No comorbidities	There are no other comorbidities.
	Kausch-Whipple operation with curative intent due to a pancreas CA 6 months ago.	Six months ago, a Kausch-Whipple operation with curative intent was performed on the patient due to a pancreas CA.
	Solitary metastatic colon carcinoma	The patient has a solitary metastatic colon carcinoma.
	Kidney failure with need for dialysis	The patient has had kidney failure requiring dialysis for 6 months.
Neurological outcome	Inconspicuous neurological findings	The patient’s central neurological findings are normal.
	Neurological damage possible	Severe central neurological damage cannot be ruled out.
	Neurological damage certain	Severe central neurological damage is assumed to be certain.

CA, carcinoma; ECLS, extracorporeal life support; ECMO, extracorporeal membrane oxygenation; VA, venoarterial; VV, venovenous.

The vignettes were included in a 14-question electronic survey developed by the authors (full copy of the survey available on request). The survey was available online in German, French, and Italian using the software Qualtrics (Qualtrics, Provo, UT). The survey was originally developed in German and subsequently translated by a professional translation agency. The translated survey versions were assessed for congruence by native French and Italian members of the study team.

The study was presented to the cantonal research ethics committee in Zurich and was determined to not require a standard review process (BASEC-Nr. Req-2017-00217). In addition, the study was assessed by an internal ethics review (CEBES) of the Institute of Biomedical Ethics and History of Medicine/University of Zurich and by the ethics committee of the Charité–Universitätsmedizin Berlin (EA1/164/17).

### Dependent Measures

The study included 2 different and independent outcome measures: 1) the willingness to initiate ECLS and 2) the readiness to withdraw active ECLS support. Participants responded to 5 different vignettes for each of the 2 outcomes.

Each vignette was followed by the statements “From my point of view, ECLS/ECMO *should* be used in this patient” and “In my clinic, ECLS/ECLS *would* be used in this patient” in the case of initiation and by the statements “From my point of view, the ECLS/ECLS treatment *should* be discontinued” and “In my clinic, the ECLS/ECLS treatment *would* be discontinued” in the case of withdrawal. The order of the statements was presented at random between respondents to control for possible order effects. A response format ranging from 1 (*not correct at all*) to 6 (*fully correct*) was used to capture respondents’ beliefs. A 6-point response format was used that would exclude a neutral midpoint since the decision to be made is either for or against the use of ECLS; this format requires participants to take a position as is required in clinical situations.

### Independent Measures

The factors included in the vignettes were chosen based on a systematic review regarding the effectiveness of ECLS,^17^ a systematic review of available ECLS guidelines and position papers,^18^ a previous qualitative project on decision making regarding ECLS, and an interdisciplinary workshop (medicine, bioethics, statistics, sociology) with the aim of critically discussing the survey. Based on this input, 7 factors were chosen for the ECLS scenario dealing with initiation, and 8 factors were chosen for the scenario dealing with discontinuation; these factors included age,^19–23^ treatment costs,^21^ resources available to operate ECLS,^19,20^ comorbidities,^19,20,22^ neurological outcome,^19,21^ the 2 common ECLS (VA-ECLS for cardiac support v. VV-ECLS for pulmonary support), and the therapeutic goal of the treatment (bridge to recovery, bridge to transplantation, bridge to decision). For the withdrawal scenario, the duration of ECLS treatment, indications for withdrawal, and the patient’s condition were additional factors included in the vignettes (Table 1 and Table 2).

The survey was pretested with a convenience sample of *n* = 10 physicians whose practice involves ECLS. The pretest also included evaluative questions regarding the clarity of terminology, if relevant questions or answer choices were missing, if the tasks were clear, or if survey questions were leading. The survey was slightly modified based on the insights of the pretest. The clinic that participated in the pretest was not part of the final study sample.

### Study Participants

All physicians in Germany and Switzerland involved in ECLS were eligible to participate in the study. The following recruitment strategy was applied: first, in Germany, ECLS-specific OPS codes (German modification of ICPM [International Classification of Procedures in Medicine] codes) were used to identify relevant hospitals. Hospitals in Switzerland with heart surgery centers were included. The Swiss Society of Perfusion (http://www.swissperfusion.ch/) supported the identification of these centers. Based on the experience of the study team, it was determined that this search strategy would likely capture hospitals that provide pulmonary ECLS services only. Second, clinical staff in departments potentially involved in ECLS, such as cardiology, pulmonology, anesthesia and intensive care, internal medicine, and heart and thoracic surgery, from the included hospitals were identified and invited to participate if eligible. The staffs’ publicly available email addresses were collected as listed on the hospital website, located by hand searching on the Internet, or, when necessary, by composing a likely address based on the hospital’s known email format. Physicians were contacted by post if the abovementioned contact efforts failed. The potential participants were contacted either by email or by post but not by both to avoid overlap.

Physicians contacted by email received an invitation letter, a link to the survey, and a link to the informed consent documents. The participants who were contacted by post received an invitation letter that included both a link and a QR code to the survey as well as a printed version of the informed consent documents. All participants provided their informed consent on the first page of the survey. The data collection lasted from June 20 to July 17, 2019. A reminder was sent to nonrespondents 1 week after the invitation by email.

### Statistical Analysis

The total number of vignettes was reduced to 120 for initiation and 180 for withdrawal by a D-efficient resolution IV design^24^ using the SAS macro %mktex, where no main effects are confounded with each other. Furthermore, the 2-factor interactions comorbidities × neurological outcome in the initiation scenarios and ECMO duration × ECMO circuit as well as ECMO duration × criteria in the withdrawal scenarios were set to remain unconfounded. The D-efficiency^25^ of this design was 98.47 for initiation and 98.95 for withdrawal. The 120/180 vignettes were then distributed to 24/36 unique vignette samples, so-called decks of vignettes, with 5 vignettes each, using the SAS macro %mktblock (see Supplemental Tables S1 and S2 for correlation matrices of the factors). From the possible sets of efficient designs, the unrealistic combination of an 80- or 90-year-old patient bridged to transplant (see below) was excluded in advance.^26^ Before answering the vignette modules of the survey, the respondents were randomly and evenly distributed to one of these decks (see Supplemental Figure S1 for the deck distributions).

The units of analysis were the vignettes and not the individual participants. To take the hierarchical data structure into account, mixed-effects regression models were fitted. The experimental design was optimized for the estimation of 7 initiation and 8 withdrawal main effects and 1 (initiation: comorbidities and neurological outcome) and 2 (withdrawal: days on ECLS and ECLS circuit; days on ECLS and criteria for withdrawal) interaction terms. The model included fixed effects for the main effects and the interactions, as well as the country (2 levels) as the blocking factor. Random effects were estimated for respondents and hospitals. Since we detected nonnormally distributed residuals, robust standard errors were calculated. Likelihood ratio test was used to assess whether the interaction terms were required. Predictive margins were calculated as postestimations based on mixed-effects models for interaction terms. All covariates were entered as dummy variables. Listwise deletion of respondents was chosen to deal with missing values. Outcome variables were treated as a metric. In a sensitivity analysis, the data were reanalyzed using multilevel ordered logistic regression. There were no qualitative differences in the results, and all estimated coefficients pointed in the same direction. The results were therefore presented in a linear regression model due to simplicity of interpretation. All statistical analyses were conducted using Stata (version 16.0; StataCorp LLP, College Station, TX).

## Results

### Respondent Characteristics

**Figure 2.**
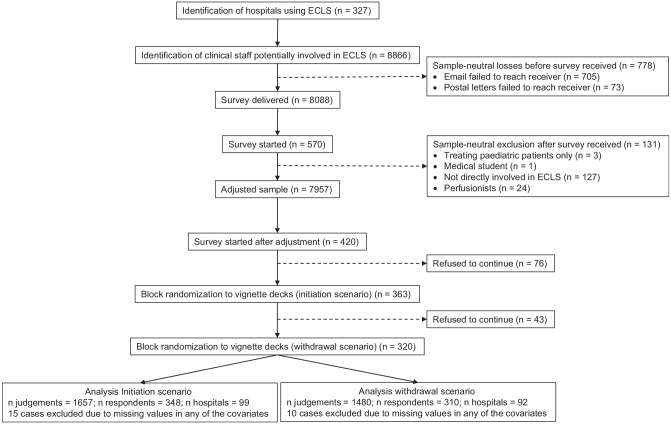
Flow diagram of study participants.

Of the 7,037 emails sent, 705 failed to reach the receiver for reasons including strong security settings and invalid initial email addresses. Among the 1,829 letters sent, 73 were returned due to invalid addresses. Respondents who indicated that they treat pediatric patients only (*n* = 3), were medical students (*n* = 1), or reported not being directly involved in ECLS (*n* = 127) were excluded before analysis. After the adjustment of sample-neutral losses, *n* = 420 physicians from *n* = 111 hospitals started the survey (Figure 2).

On average, there were 12.1 respondents within each hospital (minimum = 1; maximum = 40). The survey was completely finished by 277 respondents (66%). Among the 143 observations with item nonresponses, most participants rated all 5 vignettes in the initiation scenario (266) and the withdrawal (275) scenario. The response rate on the hospital level was 111 of 327 (33.8%). The net return rate on the respondent level was 348 of 7,956 (4.36%) for the initiation scenario and 310 of 7,956 (3.90%) in the withdrawal scenario.

Most reported more than 10 years of experience with ECLS (69/297, 23.1%), with a general mean time of working experience of 16.6 years. Respondent characteristics are shown in Table 3.

**Table 3:** Respondent Characteristics.

Characteristic	Frequency^a^ (%^b^)
Sex	
Female	63 (22.7)
Male	213 (77.2)
Years of experience with ECLS	
Less than 1 year	10 (3.5)
1–2 years	47 (17.1)
3–4 years	50 (18.2)
5–6 years	47 (17.1)
7–8 years	46 (16.6)
9–10 years	23 (8.4)
More than 10 years	52 (18.8)
Career stage	
Resident	34 (13.9)
Fellow	38 (13.4)
Junior attending physician	119 (45.8)
Senior physician	52 (17.9)
Head of department	32 (8.8)
Years of working experience, mean	16.8

ECLS, extracorporeal life support.

a Totals may not equal 420 due to missing values.

b Totals may not equal 100% due to missing values.

### Vignette characteristics

**Figure 3:**
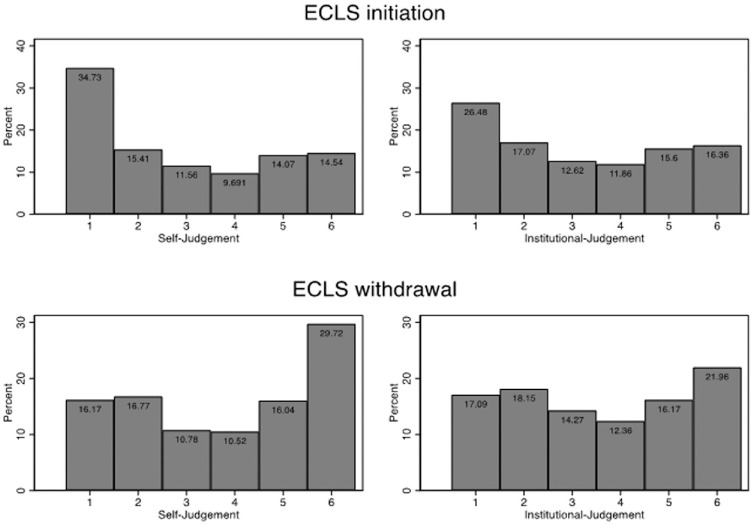
Distribution of judgments across all vignettes. Initiate ECLS: “From my point of view, ECLS/ECLS should be used in this patient” and “In my clinic, ECLS/ECLS would be used in this patient.” Discontinue ECLS: “From my point of view, the ECLS/ECLS treatment should be discontinued” and “In my clinic, the ECLS/ECLS treatment would be discontinued.” Measured on a 6-point rating scale ranging from 1 (*not correct at all*) to 6 (*fully correct*). ECLS, extracorporeal life support.

In total, there were 1,713 self-ratings and 1,711 ratings for the clinics in the initiate scenario, as well as 1,521 self-ratings and clinic ratings in the withdrawal scenario. Figure 3 shows the distribution of judgments across all vignettes for both scenarios. In all vignettes, the self-judgments tended to be in favor of not initiating ECLS in the initiation scenario and discontinuing ECLS in the withdrawal scenario (Figure 3).

### Decisions to Initiate ECLS

**Figure 4:**
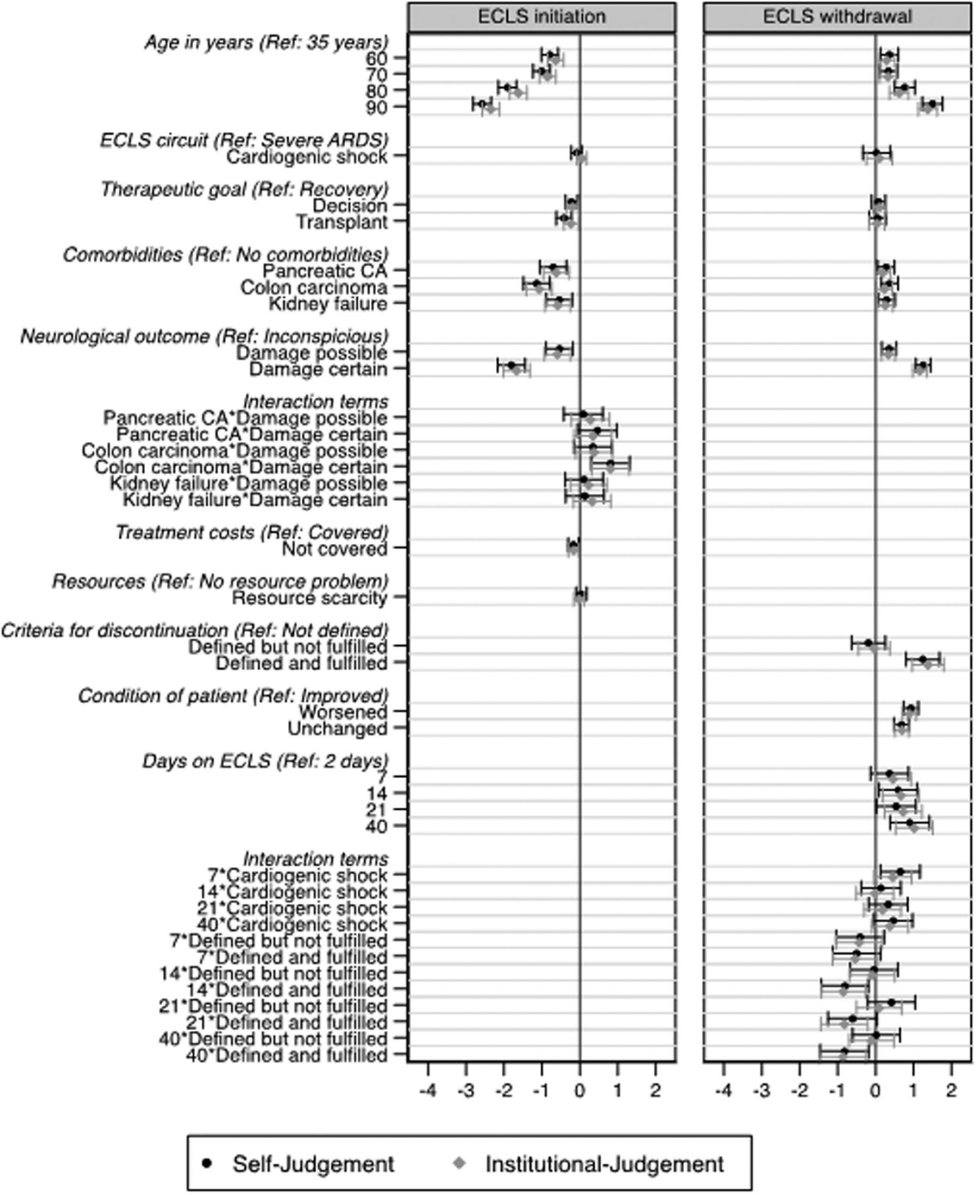
Results of mixed-effects models for self-judgments and institutional judgments. Horizontal bars indicate 95% confidence intervals. Coefficients indicate the increase/decrease of the judgments measured on a 6-point rating format, leaving all other factors unchanged. Blocking factors (not shown in the figures) are country (fixed) and hospital and respondent (both random). Results are based on 2,362 self-judgments and 2,360 institutional judgments from 489 respondents in 134 hospitals in the initiate model, as well as 2,185 self-judgments and 2,179 institutional judgments from 451 respondents in 128 hospitals (450 respondents and 127 hospitals in institutional judgments) in the withdrawal model. Likelihood ratio test, comparing the model with interaction terms with a smaller model that does not include the interaction term, yielded strong evidence that the interaction between comorbidities and neurological outcome (ECLS initiation model), as well as between days on ECLS and ECLS circuit and days on ECLS and criteria for withdrawal, should be kept in the model (initiation, *P* = 0.0018; withdrawal, *P* = 0.0047). ARDS, acute respiratory distress syndrome; CA, carcinoma; ECLS, extracorporeal life support.

**Figure 5:**
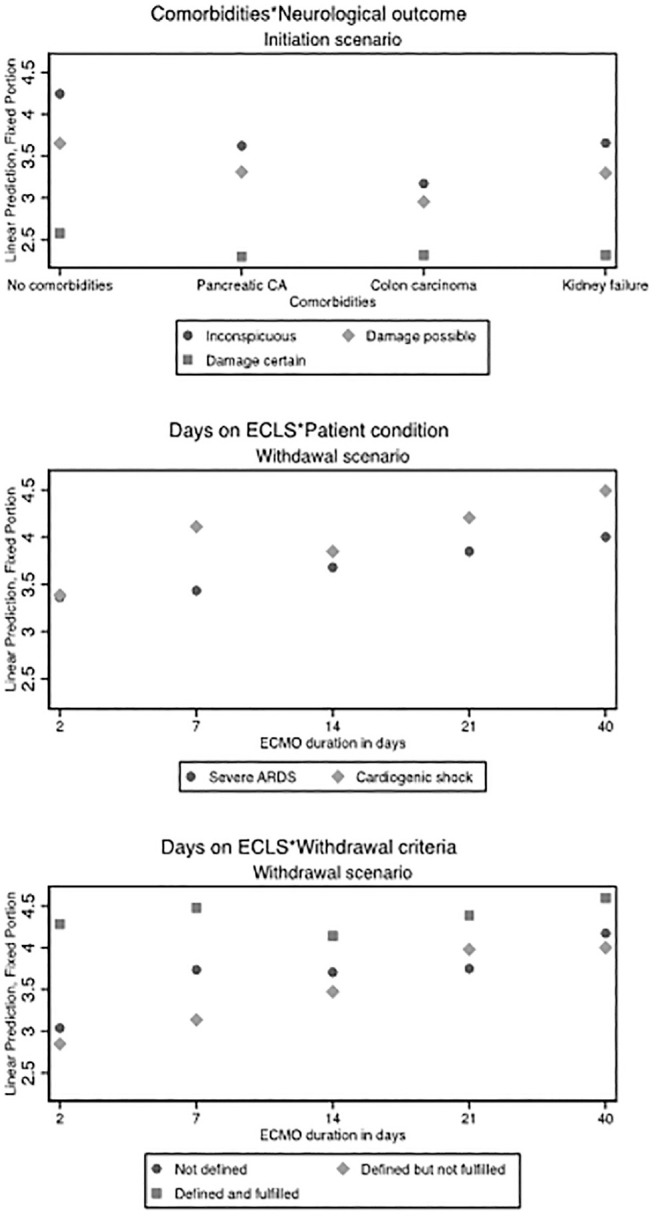
Predictive margins of ECLS judgments. The y-axis shows the predicted probabilities of the self-judgments, calculated as postestimation based on mixed-effects models. Predicted margins are calculated for specific groups (e.g., comorbidities), assigning each respondent to that group while leaving all other factors unchanged (e.g., if all respondents would have assessed vignette patients with kidney failure and a certain neurological damage, we would expect an average willingness to initiate ECLS of 1.8 scale points). ECLS, extracorporeal life support.

Figure 4 shows the analysis of vignette factors on decisions to initiate and to withdraw ECLS for self-judgments and institutional judgments. In the self-judgments, very old age (90 years) of the patient was the strongest factor influencing the vignette judgments (−2.58; 95% CI, −2.90 to −2.34; *P*<0.001). A noncoverage of treatment costs led to a small but significant decrease of self-judgments to initiate ECLS (−0.16; 95% CI, −0.29 to −0.06; *P*=0.004). The second biggest factor that decreased the initiation judgments by almost 2 scale points was certain neurological damage (−1.79; 95% CI, −2.22 to −1.38; *P*<0.001). Among possible comorbidities, colon carcinoma with solitary metastases had the strongest influence on the judgments (−1.14; 95% CI, −1.47 to −0.80; *P*<0.001). Furthermore, there was a significant positive effect of certain neurological damage in interaction with colon carcinoma (0.81; 95% CI, −0.33 to −1.29; *P*=0.001) (see also Figure 5 for predictive margins of interaction effects). Respondents were less likely to initiate ECLS in vignettes where ECLS served as a bridge to decision (−0.21; 95% CI, −0.36 to −0.06; *P*=0.004) or a bridge to transplant (−0.42; 95% CI, −0.79 to −0.24; *P*<0.001) compared to scenarios where ECLS served as a bridge to recovery. There were no significant effects based on the type of ECLS circuit or based on resource scarcity. All institutional judgments were comparable to the self-judgments.

### Decisions to Withdraw ECLS

Decisions to withdraw ECLS were significantly influenced by patients’ age; a 90-year-old patient increased the self-judgment to withdraw ECLS by 1.50 scale-points (95% CI, 1.20−1.79; *P*<0.001). The model revealed a trend related to days on ECLS such that the longer a patient was on ECLS, the more likely it was that ECLS should be withdrawn (0.90; 95% CI, 0.36−1.43; *P*=0.001). Clearly defined and fulfilled criteria for ECLS discontinuation led to a higher acceptance of ECLS withdrawal (1.23; 95% CI, 0.74−1.74; *P*<0.001). There was a greater readiness to withdraw ECLS in scenarios where the patient had been on support for longer than 7 days and had cardiogenic shock (0.64; 95% CI, 0.07−1.23; *P*=0.025).

Participants were less likely to withdraw support from day 14 in scenarios where there were defined and fulfilled criteria for discontinuation; this association slightly decreased as the number of days on ECLS increased (see also Figure 5 for predictive margins of interaction effects). However, the effect remains significant up to day 40 on ECLS. Compared to an improved patient condition on ECLS, a worsened condition increases the judgment to withdraw ECLS (0.92; 95% CI, 0.75−1.12; *P*<0.001). All included comorbidities resulted in a slight increase of judgments in favor of ECLS withdrawal. As in the initiation model, certain neurological damage was the second biggest influencing factor for withdrawal decisions (1.24; 95% CI, 1.03−1.45; *P*<0.001). Possible neurological damage, however, influenced the judgments only moderately (0.36; 95% CI, 0.16−0.53; *P*<0.001). There were no significant effects related to the type of ECLS circuit or the therapeutic goal (bridge to decision and bridge to transplant compared to bridge to recovery) on withdraw decisions. As in the initiation scenario, all institutional judgments were comparable to the self-judgments.

## Discussion

The present study highlights several key factors that influence decisions to initiate or withdraw ECLS support, including advanced age, neurological status, and comorbidities. The results reveal a strong relationship between patients’ age and decisions to initiate and withdraw ECLS: the older the patients, the more likely participants were not to initiate and to withdraw ECLS. These judgments are in line with several guidelines^19–23^ as well as with findings from a recent survey.^27^ While not all of these guidelines specify a clear age cutoff, some suggest individual deliberation of ECLS in patients >75 years.^19,20^ Increased age is associated with mortality due to acute respiratory^28^ and cardiac failure.^29^ Furthermore, patient’s age is considered in several tools to predict mortality.^30,31^

Decisions for both initiation and withdrawal of ECLS were strongly influenced by certain poor neurologic outcome but only moderately influenced in vignettes where neurological damage was only a possibility. Risk factors for neurologic damage can be divided into factors present prior to ECLS initiation and those that are related to events that occur during ECLS.^32^ This differentiation is captured in the present study by the 2 different scenarios. In clinical practice, ECLS is associated with neurological complications in approximately 10% of the patients. Reported neurological complications range from subtle cognitive impairments to seizures, strokes, and intracerebral hemorrhage, up to brain death, and are associated with higher in-hospital mortality.^32–34^ These factors might explain why the neurological condition was such an important aspect of the decision process of the respondents. Using neurological factors as a guide, though, is challenging since the assessment, management, and long-term prognostication of such complications are not straightforward.^32^

It should be emphasized that emotional, cognitive, and behavioral impairments resulting from moderate to severe brain injuries can have significant consequences on an individual’s interpersonal, social, and occupational functioning as well as on his or her capacity for independence.^35^ For the ECLS treatment team, patients for whom there are neurological risks or for whom adverse events have already occurred pose numerous ethical challenges. For example, the decision to limit life-sustaining treatments such as ECLS in patients with severe neurological damages incapable of giving informed consent can be complicated. Such decisions run the risk of leading to unwanted futile outcomes.^36^ Even the concept of futility is neither objective nor universally recognized; it is rather determined by the values of the patient, his or her family, and health care providers.^36^

An unexpected finding was that even though significant effects were found related to comorbidities for ECLS initiation and withdrawal decisions, there were no observed differences between the comorbidities: compared to a vignette patient without any comorbidities, a vignette patient with kidney failure decreases the judgment to initiate ECLS to the same degree as a vignette patient with pancreatic cancer after Whipple surgery. Furthermore, we observed a positive interaction effect between the mentioned comorbidities and possible or certain neurological damage. These findings might reflect a change in ECLS utilization. A study comparing characteristics and outcomes of ECLS patients before and after the release of the Conventional Ventilation or ECMO for Severe Adult Respiratory Failure (CESAR) trial reported that patients on ECLS after the CESAR trial were more likely to have 2 or more comorbidities than patients before the publication of the trial.^38^ The CESAR trial was the first randomized controlled trial comparing patients with respiratory failure on ECMO to conventional supportive critical care. A retrospective cohort study in New York State points in a similar direction, reporting that ECLS patients after 2009 were more likely to have major comorbidities, including chronic kidney disease and liver disease.^39^

It was initially hypothesized that longer days on ECLS in addition to defined and fulfilled withdrawal criteria would lead to an increase in withdrawal judgments. Interestingly, the opposite was observed: the longer on ECLS in combination with defined and fulfilled withdrawal criteria, the lower the intention to withdraw ECLS. Our findings may reflect a tendency to postpone withdrawal decisions of running ECLS treatments and the reluctance to “give up” after an intense investment in life-prolonging care. Such decisions may also occur in cases in which relatives wish for the treatment to continue even though it is futile^40^ or can be attributed to moral distress among health care professionals.^41^

The study suggested a small but significant negative effect of noncovered treatment costs for ECLS. In the light of rising health care costs, particularly in end-of-life care, this finding is not surprising. Similarly, Meltzer et al.^37^ found that physicians would restrict VA-ECMO given its costs. However, this may also reflect economic pressure put on ECLS practitioners.

The response rate of our study seems considerably low. In this regard, 2 aspects need to be discussed. First, it should be noted that our sampling strategy is highly affected by overcoverage, since numerous participants were contacted who are not involved in ECLS. We estimate that in fact there are approximately 10 ECLS users on average per hospital in Switzerland and Germany. This unknown extent of overcoverage has likely affected the calculation of the response rate. Second, the key factor for satisfactory factorial surveys is not the random selection of participants but rather a well-established setup of vignettes containing the experimental stimuli that are randomly allocated to the participants. If these conditions are met, valid conclusions on the causal impact of the vignette factors should not strongly be affected by coverage or nonresponse problems.^42^

The present study has several limitations. First, we selected specific factors based on theoretical assumptions while removing much real-world, medical complexity. Furthermore, the present study design measured expressed behavior and not actual behavior. Such removals of complexity are inherent to experiments in general. The main purpose of experiments in general and of factorial surveys in specific, however, is not to make generalizations of behaviors but the testing of underlying mechanisms.^16,42^ Second, the judgments may be underreported due to social desirability bias (e.g., regarding effects of resource scarcity on medical decisions). Compared to conventional survey items, this effect is usually weakened as respondents are not fully aware of the statistical effects of the different factors of complex vignettes.^26^ Third, only 2 countries were surveyed, which might have influenced the results. Finally, the overall response rate on the hospital level was 44.2%. Since the population on the respondent level is unknown, the response rate on the hospital level is the only indicator for the distribution of the survey. There is no shared consensus about acceptable response rates in surveys. In the literature, response rates between 50% and 80% are discussed as acceptable.^43^ Nonresponse rates are not directly linked to nonresponse biases.^44^ However, it is unclear in how far nonrespondents on the individual as well as on hospital level systematically differ in their response behavior.

## Conclusion

This study provided insights into physicians’ decision making processes about ECLS initiation and withdrawal. Patients’ age and neurological status were the strongest factors influencing decisions regarding initiation of ECLS as well as for ECLS withdrawal. The assessment and management of neurological complications is challenging. The findings may contribute to a more refined understanding of complex decision making for ECLS.

## Supplemental Material

sj-docx-1-mdm-10.1177_0272989X211040815 – Supplemental material for Key Factors in Decision Making for ECLS: A Binational Factorial Survey Click here for additional data file. Supplemental material, sj-docx-1-mdm-10.1177_0272989X211040815 for Key Factors in Decision Making for ECLS: A Binational Factorial Survey by Daniel Drewniak, Giovanna Brandi, Philipp Karl Buehler, Peter Steiger, Niels Hagenbuch, Sabine Stamm-Balderjahn, Liane Schenk, Ana Rosca and Tanja Krones in Medical Decision Making

sj-docx-2-mdm-10.1177_0272989X211040815 – Supplemental material for Key Factors in Decision Making for ECLS: A Binational Factorial Survey Click here for additional data file. Supplemental material, sj-docx-2-mdm-10.1177_0272989X211040815 for Key Factors in Decision Making for ECLS: A Binational Factorial Survey by Daniel Drewniak, Giovanna Brandi, Philipp Karl Buehler, Peter Steiger, Niels Hagenbuch, Sabine Stamm-Balderjahn, Liane Schenk, Ana Rosca and Tanja Krones in Medical Decision Making

## References

[1] Sidebotham D McGeorge A McGuinness S , et al. Extracorporeal membrane oxygenation for treating severe cardiac and respiratory disease in adults: part 1—overview of extracorporeal membrane oxygenation. J Cardiothorac Vasc Anesth. 2009;23:886–92. 10.1053/j.jvca.2009.08.006 19944353

[2] Del Sorbo L Fan E Nava S Ranieri VM . ECCO2R in COPD exacerbation only for the right patients and with the right strategy. Intensive Care Med. 2016;42:1830–1. 10.1007/s00134-016-4493-2 27586993

[3] Chambrun MP de Bréchot N Combes A. Venoarterial extracorporeal membrane oxygenation in cardiogenic shock: indications, mode of operation, and current evidence. Curr Opin Crit Care. 2019;25:397–402. 31116109 10.1097/MCC.0000000000000627

[4] Combes A Bréchot N Luyt C-E Schmidt M. Extracorporeal membrane oxygenation: beyond rescue therapy for acute respiratory distress syndrome? Curr Opin Crit Care. 2017;23:60–5. 10.1097/MCC.0000000000000375 27875409

[5] Abrams D Brodie D. Emerging indications for extracorporeal membrane oxygenation in adults with respiratory failure. Ann Am Thorac Soc. 2013;10:371–7. 10.1513/AnnalsATS.201305-113OT 23952860

[6] Abrams D Brodie D. Novel uses of extracorporeal membrane oxygenation in adults. Clin Chest Med. 2015;36:373–84. 10.1016/j.ccm.2015.05.014 26304275

[7] Lo Coco V Lorusso R Raffa GM , et al. Clinical complications during veno-arterial extracorporeal membrane oxigenation in post-cardiotomy and non post-cardiotomy shock: still the Achille’s heel. J Thorac Dis. 2018;10:6993–7004. 30746245 10.21037/jtd.2018.11.103 PMC6344687

[8] Extracorporeal Life Support Organization. ECLS Registry Report: International Summary. Ann Arbor, MI: Extracorporeal Life Support Organization; 2018.

[9] Quintel M Gattinoni L Weber-Carstens S. The German ECMO inflation: when things other than health and care begin to rule medicine. Intensive Care Med. 2016;42:1264–6. 10.1007/s00134-016-4380-x 27272677

[10] DeMartino ES Braus NA Sulmasy DP , et al. Decisions to withdraw extracorporeal membrane oxygenation support: patient characteristics and ethical considerations. Mayo Clin Proc. 2019;94:620–7. 10.1016/j.mayocp.2018.09.020 PMC10893957 30853261

[11] Brodie D Curtis JR Vincent J-L , et al. Treatment limitations in the era of ECMO. Lancet Respir Med. 2017;5:769–70. 10.1016/S2213-2600(17)30263-1 28705688

[12] Courtwright AM Robinson EM Feins K , et al. Ethics committee consultation and extracorporeal membrane oxygenation. Ann Am Thorac Soc. 2016;13:1553–8. 10.1513/AnnalsATS.201511-757OC PMC5059495 27299991

[13] Wolfson RK Kahana MD Nachman JB Lantos J. Extracorporeal membrane oxygenation after stem cell transplant: clinical decision-making in the absence of evidence. Pediatr Crit Care Med. 2005;6:200. 15730609 10.1097/01.PCC.0000155635.02240.9C

[14] Charlesworth M Ashworth AD Barker JM. Decision-making in response to respiratory veno-venous extracorporeal membrane oxygenation referrals: is current practice precise enough? Anaesthesia. 2018;73:154–9. 10.1111/anae.14155 29168560

[15] Rossi PH. Vignette analysis: uncovering the normative structure of complex judgments. In: Merton RK Coleman JS Rossi PH , eds. Qualitative and Quantitative Social Research: Papers in Honor of Paul F. Lazarsfeld. New York: Free Press; 1979. p 176–86.

[16] Wallander L. 25 years of factorial surveys in sociology: a review. Soc Sci Res. 2009;38:505–20.

[17] Tramm R Ilic D Davies AR , et al. Extracorporeal membrane oxygenation for critically ill adults. Cochrane Database Syst Rev. 2015;1(1):CD010381. 10.1002/14651858.CD010381.pub2 PMC6353247 25608845

[18] Brandi G Drewniak D Buehler PK , et al. Indications and contraindications for extracorporeal life support for severe heart or lung failure: a systematic review. Minerva Anestesiol. 2021;87(2):199–209. 32755087 10.23736/S0375-9393.20.14513-9

[19] Pichler P Antretter H Dünser M , et al. Positionspapier der Österreichischen Kardiologischen Gesellschaft zum Einsatz der extrakorporalen Membranoxygenation (ECMO) bei Erwachsenen kardiologischen Patienten. Med Klin Intensivmed Notfallmed. 2015;110:407–20. 10.1007/s00063-015-0052-9 26223445

[20] Beckmann A Benk C Beyersdorf F , et al. Position article for the use of extracorporeal life support in adult patients. Eur J Cardiothorac Surg. 2011;40:676–80. 10.1016/j.ejcts.2011.05.011 21683610

[21] Extracorporeal Life Support Organization. ELSO Adult Cardiac Failure Supplement to the ELSO General Guidelines. Ann Arbor, MI: Extracorporeal Life Support Organization; 2013.

[22] Extracorporeal Life Support Organization. ELSO Guidelines for Cardiopulmonary Extracorporeal Life Support. Ann Arbor, MI: Extracorporeal Life Support Organization; 2017.

[23] Deutsche Gesellschaft für Anästhesiologie und Intensivmedizin. Invasive Beatmung und Einsatz extrakorporaler Verfahren bei akuter respiratorischer Insuffizienz. Berlin AWMF; 2017.

[24] Dülmer H. Experimental plans in factorial surveys random or quota design? Sociol Methods Res. 2007;35:382–409.

[25] Kuhfeld WF Tobias RD Garratt M. Efficient experimental design with marketing research applications. J Mark Res. 1994;31:545–57.

[26] Auspurg K Hinz T Liebig S Sauer C . The factorial survey as a method for measuring sensitive issues. In: Engel U Jann B Lynn P , et al., eds. Improving Survey Methods: Lessons from Recent Research. New York and London: Routledge; 2015. p 137–149.

[27] Abrams D Pham T Burns KEA , et al. Practice patterns and ethical considerations in the management of venovenous extracorporeal membrane oxygenation patients: an international survey. Crit Care Med. 2019;47(10):1346–55. 10.1097/CCM.0000000000003910 31356471

[28] Baek MS Chung CR Kim HJ , et al. Age is major factor for predicting survival in patients with acute respiratory failure on extracorporeal membrane oxygenation: a Korean multicenter study. J Thorac Dis. 2018;10:1406–17. 10.21037/jtd.2018.03.71 PMC5906318 29707290

[29] Lee SN Jo MS Yoo K-D. Impact of age on extracorporeal membrane oxygenation survival of patients with cardiac failure. Clin Interv Aging. 2017;12:1347–53. 10.2147/CIA.S142994 PMC5576703 28883715

[30] Muller G Flecher E Lebreton G , et al. The ENCOURAGE mortality risk score and analysis of long-term outcomes after VA-ECMO for acute myocardial infarction with cardiogenic shock. Intensive Care Med. 2016;42:370–8. 10.1007/s00134-016-4223-9 26825953

[31] Schmidt M Zogheib E Rozé H , et al. The PRESERVE mortality risk score and analysis of long-term outcomes after extracorporeal membrane oxygenation for severe acute respiratory distress syndrome. Intensive Care Med. 2013;39:1704–13. 10.1007/s00134-013-3037-2 PMC7094902 23907497

[32] Xie A Lo P Yan TD Forrest P. Neurologic complications of extracorporeal membrane oxygenation: a review. J Cardiothorac Vasc Anesth. 2017;31:1836–46. 10.1053/j.jvca.2017.03.001 28625752

[33] Sutter R Tisljar K Marsch S. Acute neurologic complications during extracorporeal membrane oxygenation: a systematic review. Crit Care Med. 2018;46:1506–13. 10.1097/CCM.0000000000003223 29782356

[34] Lorusso R Gelsomino S Parise O , et al. Neurologic injury in adults supported with veno-venous extracorporeal membrane oxygenation for respiratory failure: findings from the Extracorporeal Life Support Organization database. Crit Care Med. 2017;45:1389–97. 10.1097/CCM.0000000000002502 28538440

[35] Mayo CD Scarapicchia V Robinson LK Gawryluk JR. Neuropsychological assessment of traumatic brain injury: current ethical challenges and recommendations for future practice. Appl Neuropsychol Adult. 2019;26:383–91. 10.1080/23279095.2017.1416472 29313718

[36] Stretti F Klinzing S Ehlers U , et al. Low level of vegetative state after traumatic brain injury in a Swiss academic hospital. Anesth Analg. 2018;127:698–703. 29649031 10.1213/ANE.0000000000003375

[37] Meltzer EC Ivascu NS Stark M , et al. A survey of physician attitudes toward decision-making authority for initiating and withdrawing VA-ECMO: results and ethical implications for shared decision-making. J Clin Ethics. 2016;27:281–9. 10.2217/bmm.10.117 PMC5735424 28001135

[38] Mao J Paul S Sedrakyan A. The evolving use of ECMO: the impact of the CESAR trial. Int J Surg. 2016;35:95–9. 10.1016/j.ijsu.2016.09.081 27664558

[39] Batra J Toyoda N Goldstone AB , et al. Extracorporeal membrane oxygenation in New York State. Circ Heart Fail. 2016;9:e003179. 27940495 10.1161/CIRCHEARTFAILURE.116.003179

[40] Mulaikal TA Nakagawa S Prager KM. Extracorporeal membrane oxygenation bridge to no recovery. Circulation. 2019;139:428–30. 10.1161/CIRCULATIONAHA.118.036304 30664369

[41] Williams SB Dahnke MD. Clarification and mitigation of ethical problems surrounding withdrawal of extracorporeal membrane oxygenation. Crit Care Nurse. 2016;36:56–65. 27694358 10.4037/ccn2016504

[42] McDermott R. Experimental methodology in political science. Polit Anal. 2002;10:325–42.

[43] Cummings SM Savitz LA Konrad TR. Reported response rates to mailed physician questionnaires. Health Serv Res. 2001;35:1347–55. PMC1089194 11221823

[44] Groves RM. Nonresponse rates and nonresponse bias in household surveys. Public Opin Q. 2006;70:646–75.

