# Supplementary material for: Key Factors in Decision Making for ECLS: A Binational Factorial Survey
Source: Med Decis Making. 2021 Oct 23;42(3):313–25. doi: 10.1177/0272989X211040815 (PMC8918869; doi:10.1177/0272989X211040815)
Supplement: sj-docx-1-mdm-10.1177_0272989X211040815 – Supplemental material for Key Factors in Decision Making for ECLS: A Binational Factorial Survey [file sj-docx-1-mdm-10.1177_0272989X211040815.docx]

**Supplemental Table 1.**

Correlation matrix of initiation scenario factors

|  | Age | Circuit | Cost | Bridge | Resources | Comorbidities | Neurological outcome |
| --- | --- | --- | --- | --- | --- | --- | --- |
| Age | 1 |  |  |  |  |  |  |
| Condition | 0.0136 | 1 |  |  |  |  |  |
| Cost | -0.0101 | -0.0036 | 1 |  |  |  |  |
| Bridge | -0.2899 | -0.0012 | -0.0003 | 1 |  |  |  |
| Resources | -0.0523 | 0.0358 | -0.0262 | -0.0048 | 1 |  |  |
| Comorbidities | -0.0328 | 0.0095 | -0.0013 | 0.0251 | 0.0120 | 1 |  |
| Neurological outcome | -0.0036 | -0.0112 | 0.0169 | 0.0335 | -0.0093 | 0.0020 | 1 |
| Spearman’s correlation coefficients. N=2481 | | | | | | | |

**Supplemental Table 2.**

Correlation matrix of withdrawal scenario factors

|  | Age | Days | Circuit | Criteria | Patient condition | Bridge | Comorbidities | Neurological outcome |
| --- | --- | --- | --- | --- | --- | --- | --- | --- |
| Age | 1 |  |  |  |  |  |  |  |
| Days | -0.0065 | 1 |  |  |  |  |  |  |
| Condition | -0.0038 | -0.0053 | 1 |  |  |  |  |  |
| Criteria | -0.0144 | 0.0072 | 0.0021 | 1 |  |  |  |  |
| Patient condition | -0.0093 | 0.0061 | -0.0070 | -0.0132 | 1 |  |  |  |
| Bridge | -0.2807 | -0.0309 | -0.0298 | 0.0160 | 0.0060 | 1 |  |  |
| Comorbidities | -0.0215 | 0.0107 | -0.0047 | 0.0000 | -0.0238 | 0.0142 | 1 |  |
| Neurological outcome | -0.0087 | -0.0053 | 0.0124 | -0.0006 | -0.0098 | -0.0112 | -0.0070 | 1 |
| Spearman’s correlation coefficients. N=2278 | | | | | | | | |
