# Supplementary material for: Key Factors in Decision Making for ECLS: A Binational Factorial Survey
Source: Med Decis Making. 2021 Oct 23;42(3):313–25. doi: 10.1177/0272989X211040815 (PMC8918869; doi:10.1177/0272989X211040815)
Supplement: sj-docx-2-mdm-10.1177_0272989X211040815 – Supplemental material for Key Factors in Decision Making for ECLS: A Binational Factorial Survey [file sj-docx-2-mdm-10.1177_0272989X211040815.docx]

**Supplemental Figure 1.**

Distribution of vignette decks among respondents
